# US medical school curriculum on opioid use disorder—a topic review of current curricular research and evaluation of winning student-designed opioid curricula for the 2021 Coalition on Physician Education in Substance Use Disorders curricular competition

**DOI:** 10.3389/fpain.2023.1257141

**Published:** 2023-10-27

**Authors:** Ling Cao, Jennifer Van Deusen

**Affiliations:** ^1^ Department of Biomedical Sciences, University of New England College of Osteopathic Medicine, Biddeford, ME, United States; ^2^ The Coalition on Physician Education in Substance Use Disorders (COPE), Bath, ME, United States

**Keywords:** opioid use disorder, addiction, chronic pain, medical school, curriculum, medical student

## Abstract

The opioid crisis in the US severely affected and continues to affect population's health. The opioid crisis was in part fueled by inadequate pain management, which is in part due to the inadequate education in both pain and opioid use disorder (OUD) for health care professionals. In 2021, the Coalition on Physician Education in Substance Use Disorders (COPE) organized a curricular competition soliciting US medical students-designed OUD-related curricula. Twelve winning curricula were identified. Here, we first conducted a topic review regarding current US medical school OUD curricula. Then we evaluated the COPE winning curricula and compared them to the curricula identified in the topic review. For the topic review, ten relevant databases were searched up to December 31, 2021 using a combination of pre-determined keywords. Total of 25 peer-reviewed articles were selected based on the pre-determined criteria, which included 5 articles describing opioid curricular development at the state level (AZ, CA, MA, PA, and RI), 17 research articles evaluating a curriculum developed in a single institution, 2 literature reviews, and 1 article detailing curricular development and validation processes in a single institution. Although vary in organizations and formats, state-level curricula were comprehensive and could be adopted by other states or institutions with necessary local issue-based modifications. Faculty development and critical resources were major challenges for curricular implementation. The 17 research articles exhibited good scientific quality (Medical Education Research Study Quality Instrument (MERSQI) score = 11.94 ± 2.33 (maximal score = 18)). All research articles reported to some extent, the success of respective curriculum, in improving students' knowledge in and/or attitude towards OUD, based on primarily pre- and post- comparisons. Compared to these published curricula, winning students-designed curricula had more specific focuses, diverse learning activities, and varieties in assessment methods. For all curricula, long-term evaluations were lacking. Except for the state level curricula, majority of the other curricula did not emphasize specifically on chronic pain education or the biopsychosocial approach. Interprofessional education approach was also lacking. Our topic review and curricular evaluation highlighted the needs for integrating OUD and chronic pain medical curricula, developing long-term assessment tools, and more OUD curriculum research overall.

## 1. Introduction

Opioid use disorder (OUD), a chronic relapsing mental disorder affecting ∼16 million people worldwide and over 2 million people in the US (1), is defined as a problematic pattern of opioid use that leads to significant impairment or distress (2), which can involve misuse of prescribed opioids and use of diverted opioid medications or illicit opioids (such as heroin) (3). The opioid crisis in the US severely affected and continues to affect population's health. In the US, the OUD epidemic is estimated to have an annual economic cost of over one billion dollars (4) and has caused more than a half million opioid-overdose deaths from 1999 to 2020 without a sign of slowing down (5). The most recent data estimated a total of 80,816 opioid-overdose deaths in 2021 in the US (6). Opioid-overdose related death continued to contribute to the reduction of life expectancies observed in the US despite the COVID-19 pandemic (7). It is well known that the first wave of opioid-overdose death was largely driven by the increased opioid prescription during the 1990s, which led to the publication of the guidelines for prescribing opioids for chronic pain as opioids are most often prescribed to treat pain (8). Opioid crisis also raised the concern of physician training regarding OUD and opioid management (9–11). As the result, the development of OUD curricula for medical school training has been increased. Yet the evaluation of these curricula remains incomplete and a crucial task. Thus, in this study, we first conducted a topic review regarding current research on US medical school curriculum on OUD. The involvement of chronic pain-related topics in these reported OUD curricular was also explored. Further, the Coalition on Physician Education in Substance Use Disorders (COPE) is a voluntary organization with a mission on training physician to manage substance use disorders. In 2021, COPE organized its first curricular competition soliciting US medical students (allopathic and osteopathic medical students)-designed OUD-related curricula. Twelve winning curricula were identified. Therefore, in the second part of this report, we also evaluated the COPE winning curricula and compared them to the curricula identified in the topic review which were primarily designed by the educators. Through our results, we hope to raise further awareness of the strengths and weaknesses regarding the development and evaluation of OUD-related medical school curricula.

## 2. Materials and methods

### 2.1. Literature review of curricula on OUD in US medical schools

We followed the previously published general guidelines for systematic reviews (12–14) to conduct our literature review wherever it is applicable to ensure a non-biased literature selection and review process.

#### 2.1.1. Review objectives and inclusion/exclusion criteria

The overall objective of the literature review was “Review the current literatures regarding medical education curriculum on OUD in US medical schools”. Before searching for eligible articles, we established the following eligibility criteria. Inclusion criteria: (1) Peer-reviewed full reports/articles; (2) In the format of systematic review, guideline, or research study; (3) Described the curriculum items used in the US MD or DO medical schools; (4) Related to undergraduate medical student education; and 5) Could involve students of other health professions, i.e., interprofessional or medical profession only. Exclusion criteria: (1) Abstract/poster presentations, short editorials, opinions, commentary, or individual views; (2) Reports that did not involve medical students (e.g., the program for medical residents); (3) Reports that were not related to opioids; (4) Reports that described specific one-time non-curricular activity/event, i.e., event that was not intended to be added to existing medical school curriculum; and (5) Reports that did not involve US medical schools (MD or DO).

#### 2.1.2. Identification of articles for review

The literature search was conducted with the following key words: *medical curriculum, medical student, substance use disorder, addiction medicine, opioids, used simultaneously*. The following databases were used in the literature search: (1) AccessMedicine (provider: McGraw. Hill), (2) APA PsycINFO [provider: Elton B. Stephens Company (EBSCO)], (3) CINAHL Plus® with Full Text (provider: EBSCO), (4) Clinical Key (provider: Elsevier), (5) Education databases (provider: ProQuest Information and Learning Company), (6) Education sources (provider: EBSCO), (7) ERIC (provider: EBSCO), (8) PubMed [provider: United States National Library of Medicine (NLM)], (9) Scopus (provider: Reed Elsevier), and (10) Teacher Reference Center (provider: EBSCO). Except for PubMed, the author used the library resources from the Johns Hopkins University to help identify relevant databases (such as those databases in topic areas of “Education” and “Education & Health Sciences”) to conduct the search. For all databases, all available resources up to December 31, 2021 were included in the search. Within each database, abstract-only items (such as conference poster presentations) were excluded first before downloading the identified items. All saved items were further screened to remove duplicated items. The abstracts of the remaining items were screened again based on the pre-determined inclusion/exclusion criteria. Then, full-text articles of all remaining items were obtained and reviewed in detail, followed by further selection of eligible articles for literature review based on the pre-determined inclusion/exclusion criteria. During the process of obtaining the full articles, “Similar articles” function within PubMed was also used to help identify potential additional articles. These potential additional articles were also reviewed based on the same inclusion/exclusion criteria described earlier.

#### 2.1.3. Data extraction and summary report of the identified articles

All identified articles are subjected to further data extraction using the Excel program (Microsoft office Professional Plus 2019, version 1808). Each article was assigned with a numeric ID to be used during the review process. The following items are extracted from each original article: Authors, Year of publication, Title of the article, Journal/issue/pages, Article type: Review/guideline development/research study, Study methods, Objectives, Training targets, Training topics, Training format, Assessment (outcome measures), Involvement of interprofessional/interdisciplinary students, Outcomes, and Author-identified limitations. Articles were further grouped based on the article types and separate analysis were conducted within the same types of the articles. For research studies, Medical Education Research Study Quality Instrument (MERSQI) (15) was used to assess the quality of each of the study.

### 2.2. Evaluation of COPE 2021 winning student curricula on OUD

#### 2.2.1. Identification of winning curricula for evaluation

During the spring of 2021, COPE announced a call for submissions to *the Medical Student Curriculum Challenge: Innovative Learning and Teaching About Substance Use/ Opioid Use Disorders* with the support of the Opioid Response Network (https://opioidresponsenetwork.org). COPE invited medical student individuals or teams to submit integrative curricula under the guidance of a faculty mentor. Among the 36 curricula received, 8 were identified as Winners and 4 as Honorable mentions. All of these winning curricula (12 total) are available for free downloading through COPE's web page (https://www.copenow.org/innovative-curriculum-downloads/).

#### 2.2.2. Data extraction and summary report of the winning curricula

An Excel (Microsoft) file was set up to record extracted information from each curriculum, which included: Title, School(s) or organization of origin, Topic of focus, Learners targeted, Delivery methods, Learning activities, and Assessment, as well other information such as Integration to existing curriculum, and Possibility of virtual delivery. Information were organized and presented in a series of tables, and summary text was provided in the Results.

## 3. Results

### 3.1. Literature review of curricula on OUD in US medical schools

#### 3.1.1. Article identification

**Figure 1:**
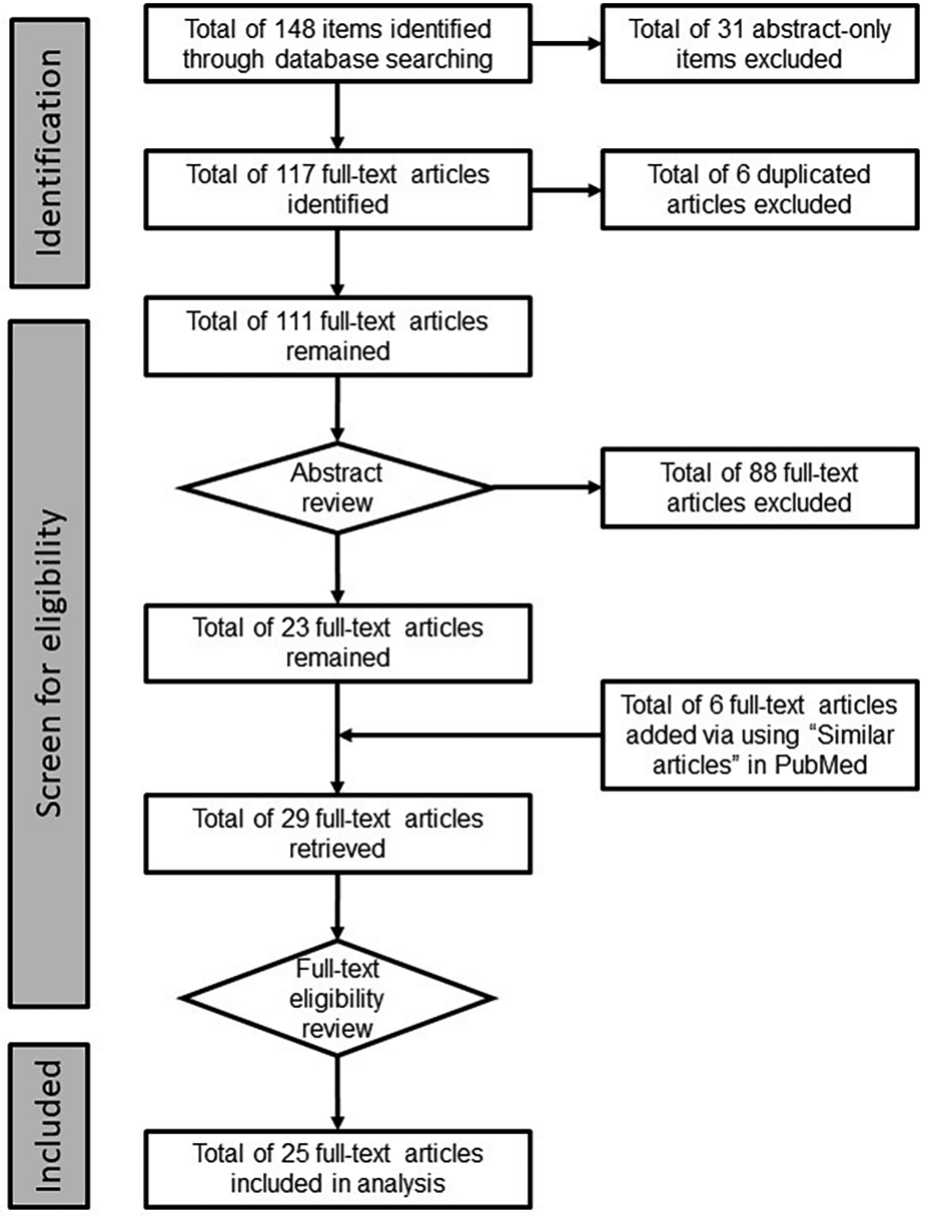
The selection process of identifying eligible articles for further analysis. Items were identified using the pre-determined key words and 10 databases as described in the Materials and methods. After removal of abstract-only items, all remaining items were subject to abstract review and subsequent full-text review based on the pre-determined inclusion/exclusion criteria. Additional articles were added via “Similar articles” function in PubMed during full-text article retrieval. Total of 25 articles were identified for further analysis.

**Figure 2:**
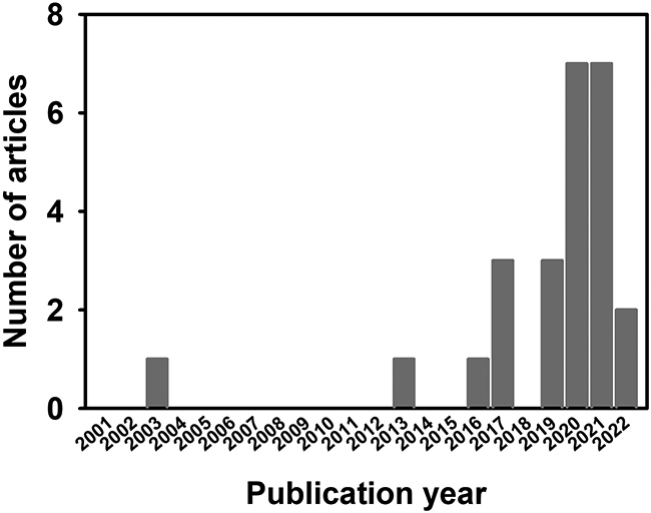
Numbers of selected articles by publication year. Numbers of articles that were selected for analysis were illustrated by their respective publication year. Majority of articles were published in 2020 and 2021 with 7 in each of these years.

Articles were first identified using the key words and databases listed in the Materials and methods. After removal of duplicated items, further selection of articles for analysis was made by applying the pre-determined inclusion/exclusion criteria (Materials and methods). The step-by-step process used in article selection was described in the Materials and methods, and are summarized in Figure 1. Total of 25 articles were selected to be used in the article analysis in this literature review, which are summarized in Table 1. Besides one article was published in 2003 (16), the rest of the selected articles were published after 2010 ranging from 2013 to 2022 (note some were available online in 2021) with most of them published in 2020 and 2021 (Figure 2).

**Table 1:** List of the 25 selected articles that were included in the analysis.

ID	Authors	Year	Title	Journal/issue/pages
1	Antman et al.	2016	Developing core competencies for the prevention and management of prescription drug misuse: A medical education collaboration in Massachusetts	Academic Medicine 91 (10): 1348–1351
2	Ashburn and Levine	2017	Pennsylvania State Core Competencies for Education on Opioids and Addiction.	Pain Med 18 (10): 1890–1894
3	Berland et al.	2017	Opioid overdose prevention training with naloxone, an adjunct to basic life support training for first-year medical students.	Substance Abuse 38 (2): 123–128.
4	Berland et al.	2019	Use of online opioid overdose prevention training for first-year medical students: A comparative analysis of online versus in-person training.	Subst Abus 40 (2): 240–246
5	Brown et al.	2013	Knowledge of addiction medicine among internal medicine residents and medical students.	Tennessee medicine: journal of the Tennessee Medical Association 106 (3): 31–33.
6	Dumenco et al.	2019	A qualitative analysis of interprofessional students’ perceptions toward patients with opioid use disorder after a patient panel experience.	Subst Abus 40 (2): 125–131.
7	Egelund et al.	2020	Recognizing opioid addiction and overdose: An interprofessional simulation for medical, nursing and pharmacy students.	Journal of Interprofessional Education & Practice 20: 100347.
8	Estave et al.	2021	Opioid stewardship training during the transition to residency to prepare medical students to recognize and treat opioid use disorder.	Subst Abus 42 (4): 1040–1048.
9	Goss et al.	2021	A Comparative Analysis of Online Versus in-Person Opioid Overdose Awareness and Reversal Training for First-Year Medical Students.	Subst Use Misuse 56 (13): 1962–1971.
10	Jennings et al.	2020	Identification and Treatment of Opioid Withdrawal and Opioid Use Disorder in the Emergency Department.	MedEdPORTAL 16: 10899.
11	Lien et al.	2021	Eight-hour medication-assisted treatment waiver training for opioid use disorder: integration into medical school curriculum.	Med Educ Online 26 (1): 1847755.
12	Monteiro et al.	2017	An interprofessional education workshop to develop health professional student opioid misuse knowledge, attitudes, and skills.	Journal of the American Pharmacists Association 57 (2): S113-S117.
13	Moore et al.	2021	Medical Student Screening for Naloxone Eligibility in the Emergency Department: A Value-Added Role to Fight the Opioid Epidemic.	MedEdPORTAL 17: 11196.
14	Moses et al.	2022	Developing and validating an opioid overdose prevention and response curriculum for undergraduate medical education.	Substance Abuse 43 (1): 309–318.
15	Moses et al.	2022	Long-term effects of opioid overdose prevention and response training on medical student knowledge and attitudes toward opioid overdose: A pilot study.	Addict Behav 126: 107172.
16	Moses et al.	2021	Training medical students in opioid overdose prevention and response: Comparison of In-Person versus online formats.	Med Educ Online 26 (1): 1994906.
17	Muzyk et al.	2019	Substance Use Disorder Education in Medical Schools: A Scoping Review	Acad Med 94 (11): 1825–1834.
18	Muzyk et al.	2020	Interprofessional Substance Use Disorder Education in Health Professions Education Programs: A Scoping Review.	Acad Med 95 (3): 470–480.
19	Riser et al.	2021	Integrating DATA 2000 waiver training into undergraduate medical education: The time is now.	Substance Abuse 42 (2): 236–243.
20	Servis et al.	2021	Responding to the Opioid Epidemic: Educational Competencies for Pain and Substance Use Disorder from the Medical Schools of the University of California.	Pain Med 22 (1): 60–66.
21	Spangler et al.	2020	Opioid Use Disorder and Assessment of Patient Interactions Among Family Medicine Residents, Medical Students, and Physician Assistant Students.	MedEdPORTAL: the journal of teaching and learning resources 16: 11012.
22	Villarroel et al.	2020	Pain and Addiction: Creation of a Statewide Curriculum. Public health reports	Public health reports (Washington, DC: 1974). 135 (6):756–762.
23	Wallace et al.	2020	Developing an opioid curriculum for medical students: A consensus report from a national symposium.	Substance Abuse 41 (4): 425–431.
24	Welsh	2003	OD's and DT's: Using movies to teach intoxication and withdrawal syndromes to medical students.	Academic Psychiatry 27 (3): 182–186.
25	Zerbo et al.	2020	DATA 2000 waiver training for medical students: Lessons learned from a medical school experience.	Subst Abus 41(4): 463–467.

#### 3.1.2. Quality review of selected articles

The 25 articles selected for analysis can be divided into 4 categories: (1) Curriculum development at state level—5 articles, IDs 1 (MA), 2 (PA), 20 (CA), 22 (AZ), and 23 (RI) (Table 1). (2) Evaluating a particular curriculum at a single institution in a research study—17 articles, IDs 3–13, 15–16, 19, 21, and 24–25 (Table 1). (3) Scoping reviews—2 articles, IDs 21 and 22 (Table 1). And (4) Curriculum development and validation at institutional level—1 article, ID 14 (Table 1).

For articles that describing the curricular development at the state level (17–21), due to the descriptive nature, no quality review was conducted. Comments regarding the process and content of each curriculum are included in the analysis. The two scoping review articles (22, 23) were from the same research group and were written by adhering to the Preferred Reporting Items for Systematic Reviews and Meta-Analyses Extension for Scoping Reviews guidelines (24) with PROSPERO (https://www.crd.york.ac.uk/prospero/) registration numbers as CRD42018098874 and CRD42018098876, respectively. In the one article that described the detailed process of curricular development and validation in a single institution (25), the authors used the published curriculum development approach (26) to develop and validate their curriculum. This curriculum was further evaluated for its efficacy in subsequent studies that were included in the 17 research study articles used in this review (27, 28). Therefore, the specific quality review was not conducted for the original curricular development article (25).

For the 17 research study articles that evaluated respective OUD curricula at institutional level, we conducted the quality review using the established instrument, MERSQI (15). The evaluation results are summarized in Table 2 with a total MERSQI score at 11.94 ± 2.23 (mean ± SD) out of the maximum possible score of 18.

**Table 2:** Summary of quality review of research studies using the MERSQI^a^.

Domain	MERSQI item	Possible score	Mean (SD) domain score	Number (%) of 17 studies
Study design	***1. Study design***		*1.59* (*0.51)*	
	Single-group cross-sectional or single-group posttest only	1	* *	4 (23.53)
	Single-group pre- and posttest	1.5	* *	8 (47.06)
	Nonrandomized, 2 groups	2	* *	4 (23.53)
	Randomized controlled trial	3	* *	1 (5.88)
Sampling	***2. Institutions***		*0.50* (*0.00)*	
	Single institution	0.5	* *	17 (100.00)
	Two institutions	1	* *	0 (0.00)
	More than 2 institutions	1.5	* *	0 (0.00)
	***3. Response rate***		*1.21* (*0.36)*	
	Response rate < 50% or not reported	0.5	* *	2 (11.76)
	Response rate 50%–74%	1	* *	6 (35.29)
	Response rate ≥ 75%	1.5	* *	9 (52.94)
Type of data	***4. Type of data***		*2.53* (*0.87)*	
	Assessment by study subject	1	* *	4 (23.53)
	Objective measurement	3	* *	13 (76.47)
Validity of evaluation instruments’ scores	***5. Content***		*1.00* (*0.00)*	
	Not reported or not applicable	0	* *	1 (5.88)
	Reported	1	* *	16 (94.12)
	***6. Internal structure***		*0.63* (*0.50)*	
	Not reported or not applicable	0	* *	7 (41.18)
	Reported	1	* *	10 (58.82)
	***7. Relationships to other variables***		*0.63* (*0.50)*	
	Not reported or not applicable	0	* *	7 (41.18)
	Reported	1	* *	10 (58.82)
Data analysis	***8. Complexity of analysis***		*1.59* (*0.51)*	
	Descriptive analysis only	1	* *	7 (41.18)
	Beyond descriptive analysis	2	* *	10 (58.82)
	***9. Appropriateness of analysis***		*0.94* (*0.24)*	
	Data analysis inappropriate for study design or type of data	0	* *	1 (5.88)
	Data analysis appropriate for study design or type of data	1	* *	16 (94.12)
Outcomes	***10. Outcomes***		*1.47* (*0.45)*	
	Satisfaction, attitudes, perceptions, opinions, general facts	1		4 (23.53)
	Knowledge, skills	1.5		12 (70.59)
	Behaviors	2		0 (0.00)
	Patient/health care outcome	3		1 (5.88)
Total MERSQI score		18	11.94 (2.23)	

^a^ MERSQI scores for individual articles are available from the corresponding author upon request.

#### 3.1.3. Information synthesis of state-level curricula

A total of 5 articles that described the development of medical school curriculum on opioid use disorder in 5 individual states: Arizona (AZ) (20), California (CA) (17, 19), Massachusetts (MA), Pennsylvania (PA), and Rhode Island (RI) (21). The general information regarding curricular development process, structure of the final products, and coverage on pain are summarized in Table 3.

**Table 3:** Summary of opioid curricular development process at the state level.

State^a^	MA	PA	AZ	RI	CA
Initiator	State	State	State	The single RI medical school	The six UC medical schools
Working group composition	Medical education working group: Medical school Deans and faculty, Leaders from MA Department of Public Health and MA medical Society	Pennsylvania Physician General task force: Representatives from all PA allopathic and osteopathic medical schools and various state and federal governmental agencies	Voluntary working group: Deans and curricular representatives from all 18 AZ health programs co-chaired by one individual from Arizona Department of health and Services and one from Phoenix VA Health Care System	Symposium participants at the opioid curricular development breakout group: 33 educators representing 14 health professional institutions from 14 states	Opioid crisis workgroup: representatives from all UC medical schools
Working time period	2015 October-December	2015–2016	2018 January - July	2018 June 10–12	2018–2019
Sources of information	Literature review, current medical school practice and existing curricula (the 4 schools), and national and local standards for treating SUD	Literature review and survey of graduating medical students	Existing federal level guidelines/reports, other state-level curricula, licensing board requirements, and professional competencies	Input from symposium participants: 113 Professionals from 23 states and 30 institutions including individuals served at state and federal levels, as well as patients and students	Available resources including competencies from other states, institutions, and that in the publications, as well as existing online teaching resources
Product title	Core competencies for the prevention and management of prescription drug misuse	Core competencies on opioids and addiction	Arizona Pain and Addiction Curriculum	Opioid curriculum and core competencies	University of California pain and substance use disorder competencies
Structures	10 core competencies organized into three domains: Primary (3), Secondary (3), and Tertiary (4) prevention domains	9 core domains with specific competencies listed within each domain	10 core components organized into 3 curricular areas: Redefine pain and addiction (3), Whole-person approach (4), and Systems perspective (3); with detailed objectives listed for each component.	15 core competencies organized under 3 general categories: Pain management (4), OUD (5), and Other areas-flexible items (6)	9 domains organized under 3 sections: Pain (4), SOD (4), and Public health (1), with specific competencies listed within each domain
Pain coverage	3 out of 10 core competencies with 2 in primary and 1 in secondary prevention of prescription drug misuse	6 out of 9 domains: pain assessment (1), acute pain treatment (2), and chronic pain management (3)	Pain and SUD addressed together within each core component	1 major section on pain management: pain physiology, assessment, treatment, and biopsychosocial nature	1 section with 4 domains: Multidimensional nature of pain, pain assessment, pain treatment, context of pain

^a^ States are listed in the order of the chronological time when each curriculum was developed.

Regardless whether it was the state or the medical schools who initiated the curricular development effort, in each of the 5 states, a working group was identified and available information (literature, existing guidelines and curricula, experts' opinions, and/or inputs from patients and students) was sought after and utilized to generate the final product. Besides the comprehensive components regarding SUD or OUD, all of these teams recognized the importance of addressing pain components in their respective final competencies/curricula (AZ developed a curriculum while others developed core competencies; Table 3). The document presented by RI, CA and AZ also had special emphasis on the biopsychosocial approaches to both pain and OUD. Further, SUD/OUD were viewed through the public health lens and public health components were included in all states' competencies/curricula. In addition, all states' competencies/curricula emphasized evidence-based practice. MA was the first state taking on this challenge, whose product was reviewed/referenced during the curricular development in other states. CA's competencies are the most updated as it was developed later and referenced materials from other states. AZ developed a comprehensive curriculum for all health professions, which could help to facilitate the collaboration and communications among all health professions in future practice as the curriculum provides the common language and concepts across all professions.

Regarding curricular delivery, while details in MA and PA were not described, the article from RI provided general guidelines on effective delivery with suggestions of using team-based, multidisciplinary activities, reflective writing, small group discussion, and empathy training, as well as incorporation of OUD training into the training for other chronic diseases. Articles from CA and AZ described the on-going effort of developing teaching resources for educators. Article from PA mentioned the state's effort in promoting sharing among medical schools regarding implementing the new competencies.

Regarding assessment, the article from RI provided general guidelines on effective assessment suggesting competency-based, qualitative (observational or open-ended writing) and/or subjective assessments, as well as using patient outcomes during assessment and longitudinal assessments. MA and AZ established annual review and evaluations of the curricular implementation and effects on learners, while CA focused on the development of assessment for the UC Clinical Performance Exam (CPX).

Potential challenges in curricular implementation include identifying times for additional curricular items, strategies for curricular integration, faculty development, clinical resources, and funding for education research to improve future curricular development and evaluation. AZ addressed the faculty development challenge by (a) producing a 150- page *Arizona Pain and Addiction Curriculum Faculty Guide* that detailed the evidence, reasoning, and supporting content behind each objective, and (b) organizing a teaching faculty submit discussing the new curriculum.

#### 3.1.4. Information synthesis of research studies

When the 17 research articles were reviewed, we found that most curricula covered the core components of OUD: pharmacological knowledge of opioid and medications for OUD, signs and symptoms of plus risk factors for OUD, treatment of OUD, with the primary focus on basic science and clinical knowledge. Many also included additional items related to OUD, such as opioid epidemic, racial/ethnicity and disparity in opioid epidemic, social stigma towards OUD, social barriers in treatment of patients with pain and/or OUD, and relevant law and regulations. Four programs used Drug Addiction Treatment Act of 2000 (DATA 2000) waiver trainings developed either by professional organizations or in-house (29–32). However, despite the recognition of the contribution from comorbid psychiatric disorders and relevant social factors, none of these articles mentioned specifically the use of biopsychosocial approaches in their curricula. Further, only 3 out of the 17 studied curricula mentioned pain in their major topic areas and the pain topic had a limited focus on managing pain in patient with OUD (29, 30, 33). In addition, a small number of programs focused on students' clinical skills, such as team-based practice, motivational interview and application of SBIRT (Screening, Brief Intervention, and Referral to Treatment), patient screening and education (34–36).

All programs studied were standing-alone as an addition to the respective existing medical school curricula with the reported total length ranging from 0.5–2 h (8 studies) or 8–11 h (6 studies) (3 articles did not specify the length of their programs). One article only assessed the effects of the “Patient panel”, one component of a standing-alone interprofessional education workshop, on students' attitude toward patients with OUD and perceived value of an interprofessional team in managing patients with OUD (37). Most of the programs studied targeted to medical students except 3 articles (34, 37, 38) that described interprofessional education programs although some of the other programs also had non-medical students participated in the training in parallel (32, 35, 39).

OUD curricula were delivered to medical students at various training stages during the 4-year period: year 1 (5 studies), year 2 (3 studies), year 3 (5 studies), year 4 (1 study), and mixed years (years 1–4, years 1–3, or years 3 + 4) (3 studies). Although most curricula were delivered in a non-clinical setting, 2 were in the emergency department (36, 39) and 1 during internal medicine clerkship (30). Three studies compared the effectiveness of training using online vs. in-person programs (28, 40, 41).

In terms of curricular delivery, majority of the programs had didactic components with or without a combination of various other components, such as group discussion, case-based learning, simulations, patient panels. Most studies used pre- and short-term post-tests to evaluate the effectiveness of their programs, with two studies evaluated students’ responses at 12 weeks (27) and 6 months (38) after training respectively. The respond rates were much lower when longer intervals were used in the post-test. Notably, one study used reflective writing as an assessment tool after students attended patient panels (37); two studies focused on assessing learners' hands-on clinical skills (one used a simulated scenario involving an OUD case at emergency department, and one used videos involving role-playing physician-patient interactions) (34, 35); and one study assessed patient outcomes (naloxone kit uptake) following students performing patient screening and education (36).

The common limitations identified by these studies were single institution setting with selected study cohorts, using self-reported measures, and that assessments were mostly limited to pre- and post- tests or post-test only without using randomized control study design. Some studies encountered lower than optimal responding rate and small sample size.

#### 3.1.5. Additional information from other articles

The findings from the two scoping review articles on SUD education for health professional students (22, 23) emphasized the needs for increased OUD education, incorporation of first-person experience during training, and interprofessional learning. The one article that detailed the OUD curricular development within a single medical school described the curricular development and validation process in great detail and could be used as a model reference for future curricular development by others (25).

### 3.2. Evaluation of COPE 2021 winning student curricula on OUD

Table 4 lists all winning curricula including their titles and submitters' institutions/organizations. Out of the 12 winning curricula, 8 were from medical schools in the Northeast region, with 3 in New England; and 3 from NY, 1 from PA and 1 from NJ. The other 3 were from OR, FL and IL, and one submission was by the Student Osteopathic Medical Association (SOMA) Opioid Overdose Prevention Task Force. Nine winning curricula were from allopathic (MD) medical schools and two were from osteopathic (COM) medical schools.

**Table 4:** List of winning curricula.

ID	Title	School
1^b^	Bias and stigma/preparing rising physicians for encounters in SUD care	University of New England College of Osteopathic Medicine
2^b^	Build structural competence and introduce harm reduction principles	Albert Einstein College of Medicine
3^a^	Comprehensive SUD curriculum for second year medical students	Frank H. Netter MD School of Medicine at Quinnipiac University
4^a^	Flipped classroom curriculum approach to learning about substance use disorders and their treatment	Philadelphia College of Osteopathic Medicine
5^b^	Humanizing substance use	Donald and Barbara Zucker School of Medicine at Hofstra/Northwell
6^a^	Introduction to addiction medicine	Oregon Health and Science University
7^a^	LICENSE (Language, impact, communication, engagement, non-stigmatizing, effectiveness)	Renaissance School of Medicine at Stony Brook University
8^b^	Opioid overdose identification and naloxone administration training	Florida International University Herbert Wertheim College of Medicine, FL
9^a^	Opioid use disorder: online workshop	Rutgers New Jersey Medical School
10^a^	Reduce overdose deaths	Student Osteopathic Medical Association Opioid Overdose Prevention Task Force
11^a^	Reducing stigma by unmasking unconscious bias	Rush Medical College
12^a^	Substance use disorder in pregnancy	Boston University School of Medicine

^a^ Winners.

^b^ Honorable Mention.

Tables 5, 6 (Parts 1 and 2) summarize the content, curricular design, assessment and other features of all winning curricula. The ID numbers in Table 4 are used to identify each curriculum in Tables 5, 6. In contrast to the knowledge-based curricula that were in published studies (3.1), student-designed curricula appeared to be more practice-focused with special emphases on clinical knowledge and skills. Three of them (IDs 3, 4, and 10) did report a comprehensive curriculum (Table 5 Part 1). Similar to the published studies, the proposed curricula targeted learners at various levels and across all 4-years of undergraduate medical education. The time needed to complete each of the curriculum ranged from 1.25–15 h with one curriculum (ID 1) stated that the program could be flexible and did not provide estimated total time. Most of the curricula were stand-alone program, with 4 (IDs 3, 5, 7, and 9) had integration plan including 1 (ID 7) that aimed to be integrated into the entire 4-year medical school curriculum.

**Table 5:** Content, and design, and assessment of the winning curricula—part 1.

ID	Topic focused on	Learner level (year in school)	Length (h)^a^	Learning activities					
				Asynchronous self-directed	Didactic session	Small group	Case-based learning (no “patient"^b^)	Patient/community member involved session	Other(s)
1	Attitude/stigma; Patient interactions	Not specified	Flexible	Yes	No	Yes	No	Yes	Standardized patient; Simulation; Patient partner
2	Harm reduction; Patient-centered interview	Not specified	3	Yes	Yes	Yes	Yes	Yes	
3	Comprehensive in SUD + Pain and Current research	2nd	12	Yes	No	Yes	Yes	Yes	Journal club presentation
4	Comprehensive in SUD	Not specified	8	Yes	No	Yes	Yes	Yes	Role-play; Interactive game; SMART or AA meeting
5	Humanistic approach in medicine	Incoming 1st	1.25	Yes	No	No	Yes	Yes	
6	Comprehensive in SUD + Public health component	Pre-clinical	10–15	Yes	Yes	Yes	Yes	Yes	Standardized patient; Clinal shadowing
7	Social determinants of health	All levels	10	Yes	Yes (peer-teaching)	Yes	Yes	Yes	Role-play; Standardized patient; Clinical shadowing
8	Naloxone usage (in harm reduction)	Graduating 4th	8	Yes	Yes	Yes (≤25)	Yes	No	Role-play; Standardized patient; Simulation
9	Patient experiences and barriers to care	2nd and 3rd	2	Yes	Yes	No	No	Yes	
10	Student educator	Not specified	6–8	Yes	Yes	No	No	No	Students present their educational sessions
11	Stigma	4th	2	No	Yes	Yes	No	Yes	
12	SUD in pregnancy	All levels	2	Yes	Yes	Yes	Yes	Yes	

^a^ Not all curricula included pre-program student prep time.

^b^ Standardized or real patients.

**Table 6:** Content, and design, and assessment of the curricula—part 2.

ID	Assessment				Will it be integrated to existing curriculum	How to integrate?	Can it be virtual?
	Pre- and post-surveys^a^	Group debriefing	Recorded formal reflections	Other(s)			
1	Yes	Yes	Yes		No		Not specified
2	Yes	Yes	No		No		Not specified
3	Yes	Yes	No		Yes	During the 2nd year 2-week addiction medicine module	Not specified
4	Yes	No	Yes	Additional post-survey at the end of rotations; Write a plan to guide future clinical practice	No		Not specified
5	Yes	No	Yes		Yes	During 1st year orientation, right after EMT-B training	Yes
6	No	Yes	Yes	Post-program survey only; Share a resource to peers to encourage further reading and learning	No	(But completed over several weeks)	Yes
7	Yes	Yes	Yes		Yes	Entire 4-year medical curriculum	Yes
8	Yes	No	No		No		Yes
9	Yes	No	No		Yes	During 2nd year psychiatry pre-clinical block or during 3rd year psychiatric rotation	Yes
10	Not specified^b^	No	No	Generate an educational presentation	No		Yes
11	Yes	Yes	No		No		Yes
12	Yes	Yes	Yes		No	(but best to be used during clinical years when rotating in obstetrics, emergency medicine or family medicine)	Not specified

a Not all pre- vs. post-survey have the same content; some components may only be in either the pre- or the post-survey.

^b^ Using existing online training modules.

Compared to the published studies, winning curricula proposed notably diverse learning activities with the top five activities being: Asynchronous self-directed learning (11 out of 12) > Involvement of patient or community members (10 out of 12) > Small group session (9 out of 12) > Case-based learning (no standardized or real patient) (8 out of 12) = didactic session (8 out of 12). Additional activities included standardized patient (4 out of 12), role-play (3 out of 12), simulations (2 out of 12), and clinical shadowing (2 out of 12), plus 1 winning submission proposed journal club presentations and 1 proposed attending community member meetings respectively (Table 5 Part 1). However, none of the programs specifically involved inter-professional education activities, which may be due to that the curricular challenge asked to focus on medical student training.

Regarding assessment, the top three methods were Pre- and post- surveys (10 out of 12) > Group discussion and debriefing (7 out of 12) > Formal recorded reflections (6 out of 12). Similar to the published studies reviewed, long-term evaluation is lacking. In addition, the detailed descriptions of proposed assessment plans were not presented in most of the curricula.

## 4. Discussion

This reported study was conducted during the time when there has been an increasing need of OUD training in undergraduate medical education and many medical schools have been actively developing and testing their OUD curricula. We first conducted a topic review on published studies regarding OUD curricula in US undergraduate medical education and then evaluated the winning curricula in response to the call for submission for Medical Students Curriculum Challenge in 2021 by COPE.

From the published studies regarding OUD curricula in undergraduate medical education, we realized that although some studies had a special focus in their training program, for majority of the studies, the general content of the respectively described OUD curriculum included the common core components: pharmacological knowledge of opioid and medications used to treat OUD, signs and symptoms of OUD, treatment of OUD (primarily medications used to treat OUD), and risk factors for OUD. Most of these were knowledge-based curricula. Particularly, review of the published state level OUD curricula indicated that the core OUD curriculum has been well defined and established, and became increasingly comprehensive involving growing numbers of public health-related issues, as the later ones (AZ, RI and CA) have been built upon the earlier ones (MA and PA). Therefore, during any future OUD curricular development, each institution could use a state-level, experts-developed core curriculum as a guideline/starting point, while pay special attention to the locally identified critical OUD-related issues. Therefore, based on the reported challenges encountered during curricular development, it is best to allocate limited resources to be utilized to improve curricular delivery strategies, faculty development, and creation and implementation of appropriate assessment methods rather than re-invent the content. In fact, we suggest the establishment of a national-wide, easily accessible “information-hub” that could provide up-to-date resources for curricular development including but not limited to expert content, teaching materials, assessment tools, and associated strengths and limitations, which could become a one-stop shop for anyone who is interested in developing their own OUD curriculum. This hub can be created by one professional organization or several organizations together. Potential organizations include but not limited to American Psychiatric Association (APA), American Medical Association (AMA), and American Osteopathic Association (AOA). Smaller organizations such as COPE could contribute to this endeavor as well. Besides relevant private foundations and medical education institutions, additional funding could come from federal agencies such as Substance Abuse and Mental Health Services Administration (SAMHSA), Health Resources and Services Administration (HRSA), and Department of Education (DOE), Department of Veterans Affairs (VA) through appropriate grant mechanisms. Besides hosting an OUD curricular library, the hub should also be active in organizing periodic information-exchange sessions, such as webinars, curricular demonstration, annual conferences to promote information flow and communications between medical schools, clinical training sites and medical students [our review of students-generated curricula highlighted the importance of including students in curricular development (see below)], as well as help address any curricular limitations [such as the ones identified in this curricular review (see below)]. Further, it should be noted that many reports recognized the needs of an evolving OUD curriculum that matches the current status of opioid epidemic, new knowledge regarding OUD, and emerging relevant laws and regulations. Through the proposed activities, a common central “information-hub” could also help individual curricular development teams learn about necessary updates and modifications of existing OUD curricula therefore continue to improve their curricula and education. To our knowledge and based on the topic review we conducted, an “information-hub” as described is currently not available and one needs to explore extensive number of resources in order to develop an OUD curriculum.

One important distinction revealed from the curricular review is the differences between competencies vs. curricula. Particularly, most of the state-level reports (except AZ) provided core competencies. Most of the research articles and student-designed learning documents described the curricula for respective institutions. It is accepted that while curricula provides specific learning objectives, and methods for content delivery and assessment, competencies are generated based on the desired learner outcomes and serve as bases for developing curricula that suitable for individual education settings (42). Therefore, state-level competencies provide guidelines for curricular development within individual institutions. Individual institutions develop curricula to tailor their own needs and resources. It should be noted that although AZ developed OUD curricula, it provided core components and learning objectives while an optional toolbox for operationalization the curriculum, which ensure the flexibility of the curriculum to fit various health professions and individual institutions (20).

Our review showed that various methods were used to deliver OUD curriculum, while student-designed curricula proposed notably more diverse methods than what was described in the published studies. Regardless, all reviewed research studies (17 articles) reported the success of their respective curricula to some extent, particularly, in short-term knowledge gain and attitude improvement. This suggests that methods of OUD curricular delivery can be flexible and designed based on institutional resources. However, furfure studies need to be conducted to make comprehensive comparisons (ideally using randomized control study designs) of the long-term efficacy and patient outcomes between various curricular delivery strategies. In fact, many authors did identify that the lack of long-term assessment of curricular effectiveness (such as students' practice behaviors and downstream patient outcomes) was one of the limitations of their respective studies. Therefore, it is critical that resources are allocated to assist with the development, validation, and sharing of long-term assessment tools. We are happy to see that two of the articles reported outcome measures beyond immediately after the completion of their curricula (27, 38). In addition, hands-on clinical skill training and assessment were emphasized by students-designed curricula and a few studies reported their effort in this area (34–36). This is another area many of the reviewed studies identified as areas needing assessment tool development.

Our review also identified some curricular content areas that indeed need further development. One of these areas is incorporating chronic pain and its management into OUD curricula, particularly the individual institutional OUD curricula, as state-level curricula did include pain topics, particularly, AZ and CA curricula addressed pain and OUD/SUD in parallel. Although most curricula in the published studies discussed opioid use for treating chronic pain could be a risk factor of OUD, very few curricula specifically described chronic pain management related topics as part of the OUD curriculum [except three studies (29, 30, 33)]. We realize that it is possible that the majority of pain-related content may be taught elsewhere in respective medical curriculum. However, the inter-woven relationship between OUD and pain management necessitates the integration between OUD and pain curricular components when training medical students. The curricula developed in AZ and CA set up great examples in this area (19, 20). It should be noted that although MA was the first state publishing the OUD competencies for medical education, three years prior to this, teams of interprofessional experts developed a comprehensive set of pain management domains and core competencies for health profession students, which included a sub-competencies on dependence, substance use disorder, misuse, tolerance, and addiction (43). This further highlights the importance of joint effort of addressing pain and OUD in medical curricula. Further, another significant related gap was the emphasis on the biopsychosocial approach in OUD (as well as in pain). This approach was identified as focus areas in the three newer state-level curricula (RI, CA and AZ), and was not specifically mentioned in the 17 research articles. Given the complex nature of both pain and OUD, the existence of various psychiatric comorbidities and social factors associated with both pain and OUD, biopsychosocial approach offers the most comprehensive, interdisciplinary assessment and intervention for patients (44–46). The aforementioned three state-level curricula could serve as the starting point for one to further develop an OUD curriculum with an emphasis on the biopsychosocial approach (19–21).

Another area that requires further improvement is incorporating interprofessional education/practice when addressing OUD and pain in curriculum. This was found lacking in both published studies reporting institutional OUD curricula and students-designed OUD curricula, but specifically emphasized in several state-level curricula, such as RI, AZ, and CA curricula (19–21). Particularly, we applaud that AZ curriculum was designed for all health professions to use, which would provide common language in OUD and pain management for all professions, thus greatly improve the communications and collaborations between professions (20). According to the World Health Organization, “inter-professional education occurs when two or more professions (students, residents and health workers) learn with, about, and from each other to enable effective collaboration and improve health outcomes” (47). Despite that the benefits of interprofessional education are recognized by health care professionals and students, its implementation remains challenging, in terms of institutional support, organizational barriers, and faculty development (48, 49). Additional resources and administrative support, as well as creative integration strategies are critical in improving interprofessional training for better caring for patients with OUD and/or pain.

We recognized several limitations of our study. Broader and less restrictive key words and more databases could be used in literature search, which may result in more articles included in our evaluation. Medical schools outside of US could be included that may provide additional knowledge regarding OUD curricular development internationally as OUD is a global health concern (50, 51). Student-winning curricula from COPE curricular challenge do not represent all medical students regarding their preference towards OUD curricula, yet evaluation of students-designed curricula suggested students' preference of hands-on skill training. This emphasizes the advantages of involving students in curricular development, which has been reported previously from students ranging from elementary education to professional post-graduate education (52–54). Further, although influences of patients' cultural background on patient care has been mentioned in many reviewed curricula, none of the them discussed how students' cultural background could potentially affect the delivery and efficacy of a specific curriculum. Involving students' voices in curricular design may help address this issue.

In summary, our report revealed that although incorporation of pain curriculum and interprofessional education is critical, comprehensive OUD core curricula have been well-established and can be used as guidance for future development. More resources should be devoted to curricular delivery including faculty and training resource development, and long-term assessments of student and patient care outcomes and curricular efficacy (55).

## Data Availability

The original contributions presented in the study are included in the article/Supplementary Material, further inquiries can be directed to the corresponding author.

## References

[1] Chang HY Kharrazi H Bodycombe D Weiner JP Alexander GC. Healthcare costs and utilization associated with high-risk prescription opioid use: a retrospective cohort study. BMC Med. (2018) 16:69. 10.1186/s12916-018-1058-y 29764482 PMC5954462

[2] Rhihub. Defining Opioid Use Disorder (OUD) and Medication for Opioid Use Disorder (MOUD) [Online]. Rural Health Information Hub. (2021). Available: Available at: https://www.ruralhealthinfo.org/toolkits/moud/1/definition (Accessed September 26, 2023).

[3] Strain E. Opioid use disorder: Epidemiology, pharmacology, clinical manifestations, course, screening, assessment, and diagnosis [Online]. UpToDate, Inc. (2022). Available at: https://www.uptodate.com/contents/opioid-use-disorder-epidemiology-pharmacology-clinical-manifestations-course-screening-assessment-and-diagnosis (Accessed November 20, 2022).

[4] Luo F Li M Curtis F. State-Level economic costs of opioid use disorder and fatal opioid overdose — united States, 2017. MMWR Morb Mortal Wkly Rep. (2021) 70:541–6. 10.15585/mmwr.mm7015a1 33857070 PMC8344997

[5] CDC. Understanding the Opioid Overdose Epidemic [Online]. (2022). Available at: https://www.cdc.gov/opioids/basics/epidemic.html (Accessed November 20, 2022).

[6] CDC. U.S. Overdose Deaths In 2021 Increased Half as Much as in 2020—But Are Still Up 15% [Online]. CDC/National Center for Health Statistics. (2022). Available at: https://www.cdc.gov/nchs/pressroom/nchs_press_releases/2022/202205.htm (Accessed February 20, 2023).

[7] CDC. Life Expectancy in the U.S. Dropped for the Second Year in a Row in 2021 [Online]. CDC/National Center for Health Statistics. (2022a). Available at: https://www.cdc.gov/nchs/pressroom/nchs_press_releases/2022/20220831.htm (Accessed February 20, 2023).

[8] Dowell D Haegerich TM Chou R. CDC Guideline for prescribing opioids for chronic pain—united States, 2016. Jama. (2016) 315:1624–45. 10.1001/jama.2016.1464 26977696 PMC6390846

[9] Cantone RE. Why medical students need addictions training. Med Teach. (2018) 40:421–2. 10.1080/0142159X.2017.1393050 29094624

[10] Khidir H Weiner SG. A call for better opioid prescribing training and education. Western J Emerg Med. (2016) 17:686–9. 10.5811/westjem.2016.8.31204 PMC5102592 27833673

[11] Rasyidi E Wilkins JN Danovitch I. Training the next generation of providers in addiction medicine. Psychiatric Clinics of North America. (2012) 35:461–80. 10.1016/j.psc.2012.04.001 22640766

[12] Harris JD Quatman CE Manring MM Siston RA Flanigan DC. How to write a systematic review. Am J Sports Med. (2014) 42:2761–8. 10.1177/0363546513497567 23925575

[13] Khan KS Kunz R Kleijnen J Antes G. Five steps to conducting a systematic review. J R Soc Med. (2003) 96:118–21. 10.1177/014107680309600304 12612111 PMC539417

[14] Uman LS. Systematic reviews and meta-analyses. J Can Acad Child Adolesc Psychiatry. (2011) 20:57–9. 10.1007/s00787-010-0157-x 21286370 PMC3024725

[15] Cook DA Reed DA. Appraising the quality of medical education research methods: the medical education research study quality instrument and the Newcastle-Ottawa scale-education. Acad Med. (2015) 90:1067–76. 10.1097/ACM.0000000000000786 26107881

[16] Welsh CJ. OD’s and DT’s: using movies to teach intoxication and withdrawal syndromes to medical students. Acad Psychiatry. (2003) 27:182–6. 10.1176/appi.ap.27.3.182 12969842

[17] Antman KH Berman HA Flotte TR Flier J Dimitri DM Bharel M. Developing core competencies for the prevention and management of prescription drug misuse: a medical education collaboration in Massachusetts. Acad Med. (2016) 91:1348–51. 10.1097/ACM.0000000000001347 27532868

[18] Ashburn MA Levine RL. Pennsylvania State core competencies for education on opioids and addiction. Pain Med. (2017) 18:1890–4. 10.1093/pm/pnw348 28339890

[19] Servis M Fishman SM Wallace MS Henry SG Ziedonis D Ciccarone D Responding to the opioid epidemic: educational competencies for pain and substance use disorder from the medical schools of the university of California. Pain Med. (2021) 22:60–6. 10.1093/pm/pnaa399 33316051 PMC8921611

[20] Villarroel L Mardian AS Christ C Rehman S. Redefining pain and addiction: creation of a statewide curriculum. Public Health Rep. (2020) 135:756–62. 10.1177/0033354920954505 32962529 PMC7649998

[21] Wallace PM Warrier S Kahn MJ Welsh C Fischer M. Developing an opioid curriculum for medical students: a consensus report from a national symposium. Subst Abus. (2020) 41:425–31. 10.1080/08897077.2019.1635971 31314686

[22] Muzyk A Smothers ZPW Akrobetu D Ruiz Veve J Maceachern M Tetrault JM Substance use disorder education in medical schools: a scoping review. Acad Med. (2019) 94:1825–34. 10.1097/ACM.0000000000002883 31663960

[23] Muzyk A Smothers ZPW Andolsek KM Bradner M Bratberg JP Clark SA Interprofessional substance use disorder education in health professions education programs: a scoping review. Acad Med. (2020) 95:470–80. 10.1097/ACM.0000000000003053 31651435

[24] Tricco AC Lillie E Zarin W O'brien KK Colquhoun H Levac D PRISMA Extension for scoping reviews (PRISMA-ScR): checklist and explanation. Ann Intern Med. (2018) 169:467–73. 10.7326/M18-0850 30178033

[25] Moses TE Moreno JL Greenwald MK Waineo E. Developing and validating an opioid overdose prevention and response curriculum for undergraduate medical education. Subst Abus. (2022) 43:309–18. 10.1080/08897077.2021.1941515 34214397 PMC8946718

[26] Thomas PA Kern DE Hughes MT Chen BY. Curriculum development for medical education: A six-step approach. Baltimore, MD: Johns Hopkins University Press (2015).

[27] Moses TEH Chou JS Moreno JL Lundahl LH Waineo E Greenwald MK. Long-term effects of opioid overdose prevention and response training on medical student knowledge and attitudes toward opioid overdose: a pilot study. Addict Behav. (2022) 126:107172. 10.1016/j.addbeh.2021.107172 34774365 PMC8957260

[28] Moses TEH Moreno JL Greenwald MK Waineo E. Training medical students in opioid overdose prevention and response: comparison of in-person versus online formats. Med Educ Online. (2021) 26:1994906. 10.1080/10872981.2021.1994906 34727840 PMC8567883

[29] Estave PM Jacobs ML Rukstalis M Goforth J Stone SN Choi JA Opioid stewardship training during the transition to residency to prepare medical students to recognize and treat opioid use disorder. Subst Abus. (2021) 42:1040–8. 10.1080/08897077.2021.1915918 34236292 PMC10335597

[30] Lien IC Seaton R Szpytman A Chou J Webber V Waineo E Eight-hour medication-assisted treatment waiver training for opioid use disorder: integration into medical school curriculum. Med Educ Online. (2021) 26:1847755. 10.1080/10872981.2020.1847755 33222656 PMC7717470

[31] Riser E Holterman LA Maruti S Brooklyn JR Devoe SG Tompkins BJ Integrating DATA 2000 waiver training into undergraduate medical education: the time is now. Subst Abus. (2021) 42:236–43. 10.1080/08897077.2021.1903653 33821773

[32] Zerbo E Traba C Matthew P Chen S Holland BK Levounis P DATA 2000 Waiver training for medical students: lessons learned from a medical school experience. Subst Abus. (2020) 41:463–7. 10.1080/08897077.2019.1692323 32031914

[33] Brown AT Kolade VO Staton LJ Patel NK. Knowledge of addiction medicine among internal medicine residents and medical students. Tennessee Med. (2013) 106:31–3. 23544288

[34] Egelund EF Gannon J Domenico L Nobles P Motycka CA. Recognizing opioid addiction and overdose: an interprofessional simulation for medical, nursing and pharmacy students. J Interprof Educ Pract. (2020) 20:100347. 10.1016/j.xjep.2020.100347

[35] Spangler JG Shull CN Hildebrandt CA Jones KB Brewer AL Knudson MP Opioid use disorder and assessment of patient interactions among family medicine residents, medical students, and physician assistant students. MedEdPORTAL. (2020) 16:11012. 10.15766/mep_2374-8265.11012 33204836 PMC7666834

[36] Moore PQ Cheema N Follman S Celmins L Scott G Pho MT Medical student screening for naloxone eligibility in the emergency department: a value-added role to fight the opioid epidemic. MedEdPORTAL. (2021) 17:11196. 10.15766/mep_2374-8265.11196 34950768 PMC8654700

[37] Dumenco L Monteiro K Collins S Stewart C Berkowitz L Flanigan T A qualitative analysis of interprofessional students’ perceptions toward patients with opioid use disorder after a patient panel experience. Subst Abus. (2019) 40:125–31. 10.1080/08897077.2018.1546262 30810496 PMC10499010

[38] Monteiro K Dumenco L Collins S Bratberg J Macdonnell C Jacobson A An interprofessional education workshop to develop health professional student opioid misuse knowledge, attitudes, and skills. J Am Pharm Assoc (2003). (2017) 57:S113–7. 10.1016/j.japh.2016.12.069 28159503

[39] Jennings L Warner T Bacro-Duverger B. Identification and treatment of opioid withdrawal and opioid use disorder in the emergency department. MedEdPORTAL. (2020) 16:10899. 10.15766/mep_2374-8265.10899 32656320 PMC7331957

[40] Berland N Lugassy D Fox A Goldfeld K Oh SY Tofighi B Use of online opioid overdose prevention training for first-year medical students: a comparative analysis of online versus in-person training. Subst Abus. (2019) 40:240–6. 10.1080/08897077.2019.1572048 30767715 PMC7257831

[41] Goss NC Haslund-Gourley B Meredith DM Friedman AV Kumar VK Samson KR A comparative analysis of online versus in-person opioid overdose awareness and reversal training for first-year medical students. Subst Use Misuse. (2021) 56:1962–71. 10.1080/10826084.2021.1958866 34355637

[42] Gruppen LD Mangrulkar RS Kolars JC. The promise of competency-based education in the health professions for improving global health. Hum Resour Health. (2012) 10:43. 10.1186/1478-4491-10-43 23157696 PMC3543172

[43] Fishman SM Young HM Lucas Arwood E Chou R Herr K Murinson BB Core competencies for pain management: results of an interprofessional consensus summit. Pain Med. (2013) 14:971–81. 10.1111/pme.12107 23577878 PMC3752937

[44] Bevers K Watts L Kishino ND Gatchel RJ. The biopsychosocial model of the assessment, prevention, and treatment of chronic pain. US Neurol. (2016) 12:98–104. 10.17925/USN.2016.12.02.98

[45] Cheatle MD. Biopsychosocial approach to assessing and managing patients with chronic pain. Med Clin North Am. (2016) 100:43–53. 10.1016/j.mcna.2015.08.007 26614718

[46] Wiss DA. A biopsychosocial overview of the opioid crisis: considering nutrition and gastrointestinal health. Front Public Health. (2019) 7:193. 10.3389/fpubh.2019.00193 31338359 PMC6629782

[47] Gilbert JH Yan J Hoffman SJ. A WHO report: framework for action on interprofessional education and collaborative practice. J Allied Health. (2010) 39(Suppl 1):196–7. 21174039

[48] Lash DB Barnett MJ Parekh N Shieh A Louie MC Tang TT-L. Perceived benefits and challenges of interprofessional education based on a multidisciplinary faculty member survey. Am J Pharm Educ. (2014) 78:180. 10.5688/ajpe7810180 25657367 PMC4315202

[49] Lumague M Morgan A Mak D Hanna M Kwong J Cameron C Interprofessional education: the student perspective. J Interprof Care. (2006) 20:246–53. 10.1080/13561820600717891 16777792

[50] Degenhardt L Grebely J Stone J Hickman M Vickerman P Marshall BDL Global patterns of opioid use and dependence: harms to populations, interventions, and future action. Lancet. (2019) 394:1560–79. 10.1016/S0140-6736(19)32229-9 31657732 PMC7068135

[51] WHO. Opioid overdose [Online]. World Health Organization. (2021). Available at: https://www.who.int/news-room/fact-sheets/detail/opioid-overdose#:∼:text=Worldwide%2C%20about%20275%20million%20people,drug%20use%20disorders%20in%202019 (Accessed June 14, 2023).

[52] Lu CY Nguyen Q Ersin OH. Active student engagement in curriculum development. Am J Pharm Educ. (2015) 79:30. 10.5688/ajpe79230 25861111 PMC4386751

[53] Brooman S Darwent S Pimor A. The student voice in higher education curriculum design: is there value in listening? Innov Educ Teach Int. (2015) 52:663–74. 10.1080/14703297.2014.910128

[54] Jagersma J. Empowering students as active participants in curriculum design and implementation. Online Submission. (2010) ED514196:1–13.

[55] PCSS. Opioid Use Disorder: What is Opioid Addiction? [Online]. Providers Clinical Support System. (2021). Available at: https://pcssnow.org/resource/opioid-use-disorder-opioid-addiction/ (Accessed November 20, 2022).

